# Vemurafenib Treatment of Pleomorphic Xanthoastrocytoma in a Child With Down Syndrome

**DOI:** 10.3389/fonc.2019.00277

**Published:** 2019-04-12

**Authors:** Giuseppe Petruzzellis, Diletta Valentini, Francesca del Bufalo, Giulia Ceglie, Andrea Carai, Giovanna Stefania Colafati, Emanuele Agolini, Francesca Diomedi-Camassei, Tiziana Corsetti, Iside Alessi, Angela Mastronuzzi, Franco Locatelli, Antonella Cacchione

**Affiliations:** ^1^Department of Hematology, Oncology and Stem Cell Transplantation, Bambino Gesù Children's Hospital (IRCCS), Rome, Italy; ^2^Pediatric and Infectious Disease Unit, Bambino Gesù Children's Hospital (IRCCS), Rome, Italy; ^3^University Department of Pediatrics, Bambino Gesù Children's Hospital, University of Rome Tor Vergata, Rome, Italy; ^4^Neurosurgery Unit, Department of Neuroscience and Neurorehabilitation, Bambino Gesù Children's Hospital (IRCCS), Rome, Italy; ^5^Neuroradiology Unit, Department of Imaging, Bambino Gesù Children's Hospital (IRCCS), Rome, Italy; ^6^Medical Genetics Laboratory, Bambino Gesù Paediatric Hospital (IRCCS), Rome, Italy; ^7^Department of Pathology, Bambino Gesù Children's Hospital (IRCCS), Rome, Italy; ^8^Hospital Pharmacy Unit, Children's Hospital and Research Institute ‘Bambino Gesù’ (IRCCS), Rome, Italy; ^9^Neuro-oncology Unit, Department of Hematology, Oncology and Stem Cell Transplantation, Bambino Gesù Children's Hospital (IRCCS), Rome, Italy; ^10^Department of Gynecology/Obstetrics and Pediatrics, Sapienza University of Rome, Rome, Italy

**Keywords:** brain tumor, down syndrome, BRAF V600E mutation, pleomorphic xanthoastrocytoma, vemurafenib

## Abstract

Brain tumors are the most common solid neoplasms of childhood, but they are very rarely reported in children with Down Syndrome (DS), who develop more commonly different types of malignancies. In particular, we hereby report the case of an 8-years-old child with DS that presented to our attention for neurological and endocrinological issues. Brain imaging revealed the presence of a mass that was partially resected revealing a histological diagnosis of Pleomorphic Xanthoastrocytoma (PXA), a rare WHO grade II tumor extending from the diencephalic region into the surrounding brain tissue. These tumors can harbor the *BRAF* mutation p.V600E, targetable by the specific inhibitor Vemurafenib. After confirming the presence of the mutation in the tumor, the patient was treated with Vemurafenib. The treatment proved to be effective, leading to a partial response and a stabilization of the disease. Usually, in patients with DS a reduction of the dose of chemotherapeutic drugs is necessary. Vemurafenib was instead well-tolerated as the only observed adverse effect was grade I skin toxicity. This is, to our knowledge, the first case of a PXA reported in a child with DS and the first DS patient treated with Vemurafenib.

## Background

Down syndrome (DS) is the most common chromosome abnormality among live births, with an incidence of ~1 every 800 live births (1). It is characterized by a common phenotype that can result from three main types of cytogenetic abnormalities: the most common is Trisomy 21 non-disjunction, accounting for ~95% of cases; in about 3–4% of the cases, a Robertsonian translocation involving chromosome 21 is found; the less common known abnormality is represented by Trisomy 21 mosaicism (47, +21/46), responsible for the remaining 1–2% (2).

Children with DS exhibit a wide variety of medical conditions, such as cognitive impairment, dysmorphic features, congenital heart diseases, gastrointestinal abnormalities, reduction in visual acuity and accomodation, hearing loss, endocrine, and immune deficiencies (3).

Even though life expectancy in these patients has significantly increased over the last decade, children with DS still have a higher risk of neonatal and infant mortality when compared to children without DS, because of the above mentioned comorbidities (4).

One of the most severe conditions associated with DS is the development of malignant tumors, mainly acute leukemia. On the other hand, solid tumors, and, in particular, brain neoplasms are rarely reported in patients with DS, and therefore the biological behavior and natural history of these tumors are not well-described and understood. Whereas, several oncogenes have been identified as responsible for the development of acute leukemias in DS, little is known about the molecular basis of solid tumors in this population.

Pleomorphic Xanthoastrocytoma (PXA) is a rare brain tumor that most commonly affects children and young adults. The prognosis is favorable when total resection is possible, but in patients not amenable to the eradication, chemotherapy, and/or radiotherapy are the only available therapeutic options. However, these regimes rarely lead to a control of the disease and the prognosis is generally poor (5). Between 60 and 65% of grade 2 and grade 3 PXAs are *BRAF* p.V600E mutated (6, 7) and treatment with Vemurafenib, a BRAF inhibitor approved for the treatment of *BRAF*—mutated metastatic melanoma, demonstrated efficacy in several cases of primary brain tumor, including PXAs (8).

We hereby report, to the best of our knowledge, the first case of a PXA harboring a *BRAF* p.V600E mutation in a patient with DS.

## Case Presentation

An 8-year old boy with DS was referred to the DS outpatient care unit of the Bambino Gesù Children's Hospital for progressively impaired gait and signs of early puberty.

**Figure 1:**
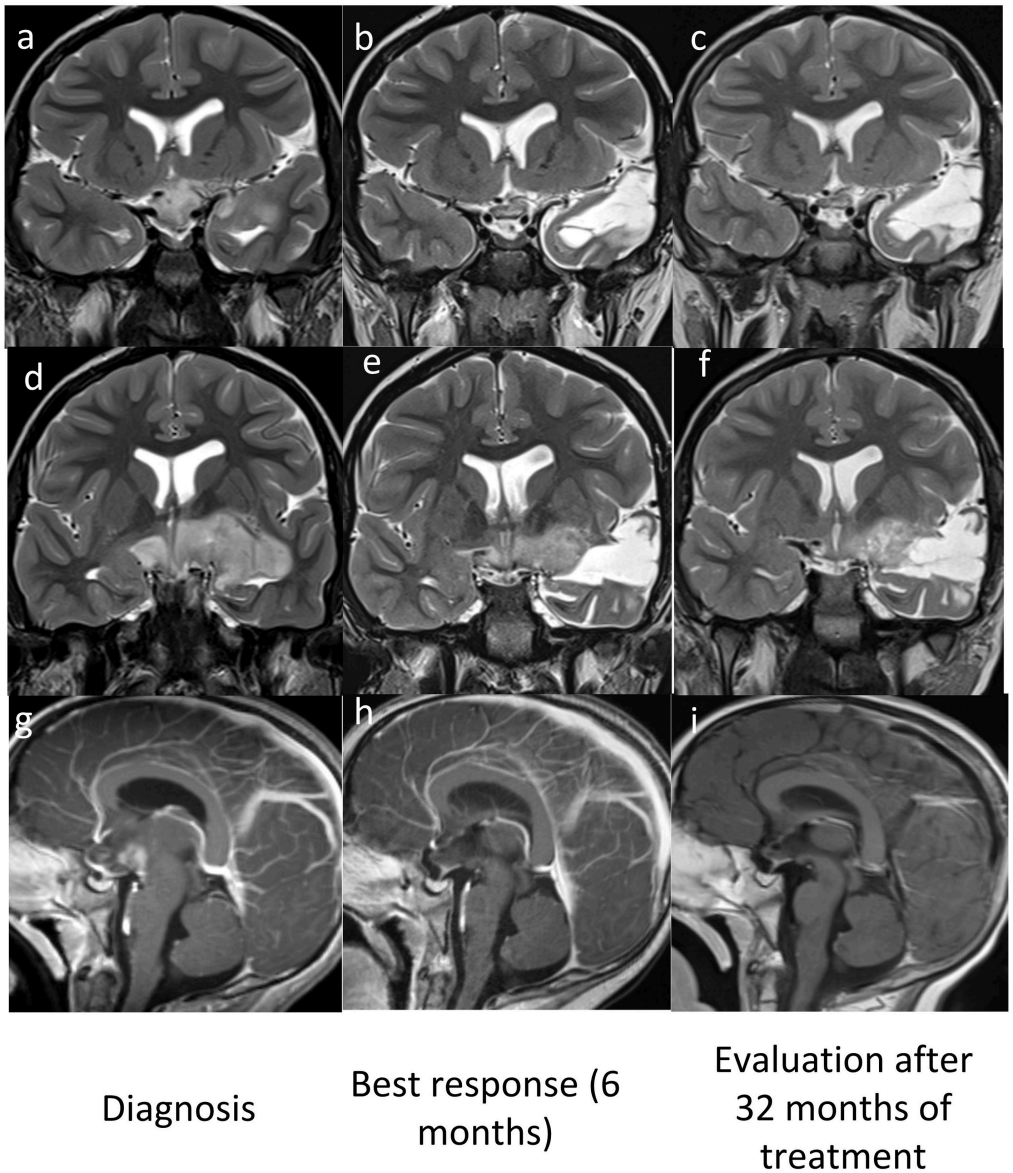
MRI pre **(a,d,g)** and post Vemurafenib **(b,c,e,f,h,i)** coronal T2w **(a–f)** and sagittal Gd T1w **(g–i)** images show best response after 6 months **(b,e,h)** and stable disease after 32 months **(c,f,i)**.

During neurological examination, a slight asymmetrical gait pattern was noted. This anomaly was firstly attributed to the general motor clumsiness typical of DS patients. When evaluating sexual development, a Tanner Stage of P2G2 was observed, with a bilateral testicular volume of 8 ml. To confirm the clinical suspect of early puberty, Gonadotropin-releasing hormone (GnRH) stimulation test was performed. The results showed: basal FSH of 0.7 mIU/mL and after LHRH administration: 3.78 mIU/mL; basal LH was 1.3 mU/mL, and after stimulation: 20.11 mU/mL; Testosterone basal level was 54.5 ng/dL, PRL, beta-HCG, DHEAS and thyroid function were all normal. These results confirmed the suspect of an early puberty of central origin and a brain Magnetic Resonance Imaging (MRI) was then performed. The brain imaging showed diffused pathological tissue, extending from the left diencephalic region and involving the cerebral peduncle caudally, the basal ganglia region cranially (globus pallidus, putamen and posterior arm of the internal capsule), the outer capsule laterally, the temporo-mesial cortex and subcortical white matter, which extended deeply to the anterior portion of the temporal lobe, to the optic chiasm and bilateral retrochiasmatic tract (Figure 1).

**Figure 2:**
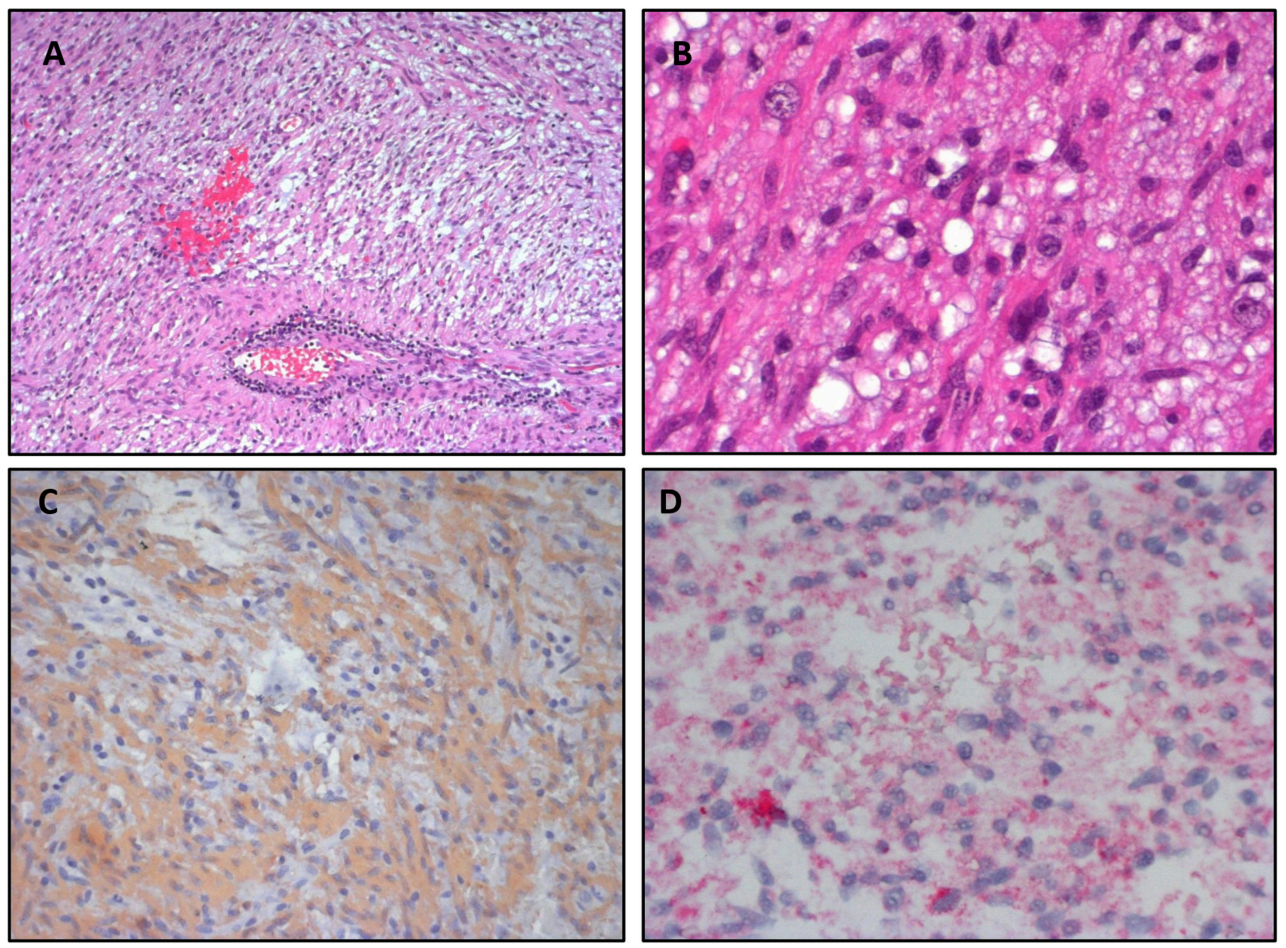
Pleomorphic Xanthoastrocytoma. **(A,B)** Hematoxylin&Eosin staining shows a marked cellular pleomorphism, with the coexistence of several cell types. **(C)** Strong positivity for GFAP in immunohistochemistry. **(D)** Positivity for BRAF V600E in immunohistochemistry.

The patient underwent partial resection of the lesion and the histopathological examination was compatible with the diagnosis of WHO grade II Pleomorphic Xanthoastrocytoma. *BRAF* p.V600E mutation was then assessed by immunohistochemistry (Figure 2) and through Sanger sequencing of the *BRAF* gene, revealing positive. Based on these results, after the parents of the patient provided formal, informed consent and the therapy was approved by Institutional Review Board, treatment with the *BRAF* p.V600E inhibitor Vemurafenib was started. Initially, the lower dose proved to be active in adults was administered (i.e., 240 mg/day *per os* twice a day) and was later increased to 480 mg twice a day. After 32 months the therapy was discontinued, and the disease remained stable 3 months after the stop therapy.

The only side effect reported was a transient follicular truncal rash in the first month of administration with fickle subcutaneous nodules, treated with local topical corticosteroids. No ECG changes or/and suspected skin lesions developed.

A new brain MRI after 6 months of therapy demonstrated an important reduction of the lesion and a substantial reduction of enhancement, the last MRI (30 months after diagnosis) demonstrated a stable disease (Figure 1).

Clinical response, with gait and movements improvement, was also noted shortly after the beginning of therapy.

## Discussion

DS is associated with several hematological disorders occurring at different ages. Neonates with DS may present with transient asymptomatic blood count abnormalities such as neutrophilia, thrombocytopenia and polycythemia. Within 1–2 months of life, 3–10% of infants with DS develop transient myeloproliferative disease (TMD) (9). Despite a spontaneous regression in most of the cases, TMD can be fatal or lead to the subsequent development of myeloid leukemia in 20% of children with DS. Children with DS also have an increased risk of developing leukemia, their risk is, in fact, approximately 10 to 20 times higher compared with children without DS (10). They represent 2% of all pediatric acute lymphoblastic leukemias (ALL) and 10% of pediatric acute myeloid leukemias (AML). The presence of somatic mutations involving GATA1 gene is associated with acute megakaryoblastic leukemia (AMKL) in people with DS (11).

In a study performed on 2,814 individuals with DS, the authors have shown that the occurrence of cancer in DS is unique with a high risk of leukemia in children and a decreased risk of solid tumors, and amongst these brain tumors, in all age-groups (12, 13).

The molecular basis of this paradox of tumorigenesis in DS is not well-understood yet. Several experimental observations have shown that DS should be a cancer-prone condition: higher rate of whole chromosome and segmental chromosome instability, increased DNA damage and defective DNA repair, immunodeficiency and susceptibility to infections, oncogenes on chromosome 21 (13). Also, abnormalities in DS related to the extra-copy of chromosome 21 include upregulation of pro-apoptotic and angiogenesis genes. For example, dysregulation of the Notch/Wnt pathway has been associated with premature aging processes in DS that may relate to leukemia (14).

On the other hand, however, cancer-inhibiting properties have been observed in other genes on chromosome 21: the *APP* gene, transcriptional factors such as ETS proto-oncogene 2 (*ETS2*), the angiogenesis suppressors Dscr1 and Dyrk1A and the *Collagen-18* gene whose fragment, endostatin, is an anti-angiogenic compound (15, 16).

Adiponectin and leptin are both involved in signaling pathways important in cancer in patients with DS. They have an opposite role, in fact leptin has many cancer-predisposing properties, such as pro-angiogenesis, reduction of apotosis, activation of Wnt, and Notch signaling (17) and it can also promote hematologic malignancies (18). Adiponectin, on the contrary, has several cancer-inhibiting properties: anti-angiogenesis, induction of apopotosis, activation of tumor-suppressors, and activation of cell signaling pathways (19–21). Several recent epidemiological studies (10, 22–27) showed that the risk of all major groups of solid tumors was decreased in patients with DS. Testicular tumors are an exception in this regard, as they seem to occur three times more often than expected in men with Down syndrome. The increased frequency of cryptorchidism (27) and testicular microlithiasis (28) may explain this evidence.

The association of DS with central nervous system tumors is extremely rare. In 1966, Miller (29) reported that among 56.199 individuals with DS only 5 developed brain tumors (estimated prevalence almost 9 per 100,000), while in the general population the estimated prevalence is 47.59/100,000 (22.31 for child, 48.49 for adolescents/young adults, and 57.75 for adult population) (30).

In the United States, brain tumors have been reported in 36 individuals with DS only, the vast majority of them represented by specific histological subgroups such as germ cells and mesenchymal tumors (13). Another study performed on post-mortem autopsies of children with DS revealed that only 3 out of 104 children presenting a malignancy had developed brain tumors (12).

One interesting example is Medulloblastoma. It is, in fact, the most common malignant brain tumor of childhood, but is particularly rare in the DS population and serves as perhaps the best example of the disparity of solid tumors in these individuals (31, 32). Similar to Medulloblastoma, Meningioma in DS was reported in only one case (33).

In this report we have described the first case, to the best of our knowledge, of Pleomorphic Xantoastrocytoma in a child affected by DS. PXA is a very rare tumor, its typical location is the temporal lobe and it usually involves both the superficial and the overlying meninges (34). Histologically, PXA is characterized by markedly pleomorphic cells, eosinophilic granular bodies, prominent reticulin deposition and a superficial meningocerebral location. PXA typically present a slow growth rate at presentation and the most common presenting symptoms are seizures. The overall survival rate is 80% at 5 years and 70% at 10 years.

Although PXAs are generally considered indolent neoplasms, they are associated with a higher frequency of recurrence, malignant transformation, and death, compared with other low-grade gliomas, such as pilocytic astrocytomas. The treatment of PXA typically involves surgical resection followed by radiological monitoring. Recurrent lesions or tumors that demonstrate anaplastic features at primary resection are treated with the same radiation and chemotherapeutic protocols used for high grade gliomas such as anaplastic astrocytoma and glioblastoma.

The mutation of the *BRAF* gene, which is typical of this tumor (7, 35), involves the activation of the MAP Kinase (MAPK) pathway, which has been shown to be the main molecular alteration present in Low Grade Gliomas (36). The most frequent mutation is the point mutation that occurs at codon 600 (*BRAF* p.V600E) that results in substitution of valine by glutamic acid.

Approximately 70% of pilocytic astrocytoma contain duplication of BRAF gene, which is rare in PXAs (37). The BRAF gene duplication leads to the formation of a fusion between the KIAA1549 locus and BRAF and the resulting protein displays a constitutively activated kinase activity, causing an aberrant activation of the downstream MAPK/ERK pathway.

Hsiao et al. found the *TMEM106B-BRAF* fusion in a case of PXA with anaplastic features in a 10-yr-old-female. This alteration results in replacement of the amino-terminal regulatory domain of BRAF with the amino-terminal region of TMEMB106B. They demonstrated that the fusion results in aberrant activation of *BRAF* signaling, with activation of MAPK/ERK pathway (38).

Inhibitors of MAPK pathway have been considered as a potential target therapy for these tumors. Among such inhibitors, Vemurafenib, a competitive small molecule that selectively recognizes the ATP binding domain of the *BRAF* p.V600E mutant, has proved to be effective in the treatment of metastatic melanoma, a neoplasm frequently associated with BRAF mutations. More recently, an activity of this drug was proved also in pediatric *BRAF* p.V600E mutated malignant astrocytomas (39).

In the case presented the mutation *BRAF* p.V600E was screened in immunohistochemistry and later confirmed by Sanger sequencing. This finding allowed us to treat the patient with Vemurafenib. This is, to our knowledge, the first case of a DS patient treated with Vemurafenib. The therapy proved to be effective as the subsequent MRI staging evaluation revealed a partial response (according to RECIST criteria) and later controls proved a stabilization of the disease.

Children with DS often respond to chemotherapy treatment as well as children without DS. However, they are more likely to experience severe toxicity with standard chemotherapy regimens, particularly those requiring methotrexate, and this often leads to a reduction of the chemotherapy doses, leading to a less effective treatment (5, 40). The treatment has been well-tolerated by the patient without serious adverse effects, proving the safety of the drug. The only adverse effect reported was grade one skin toxicity.

The use of Vemurafenib has not yet been approved for pediatric patients affected by brain tumors and DS. Although central nervous system (CNS) tumors are the most common pediatric solid organ tumor, they are very rare in patients with DS. However, the possibility of development of brain tumors in DS should be kept in mind, especially in case of unusual neurological and endocrinological symptoms. In conclusion, we have shown the safety and efficacy of Vemurafenib in a pediatric patient with DS affected by PXA.

## Ethics Statement

This study was carried out in accordance with the recommendations of the Internal Review Board of the Bambino Gesù Children's Hospital with written informed consent from all subjects. All subjects gave written informed consent in accordance with the Declaration of Helsinki. The protocol was approved by the Internal Review Board of the Bambino Gesù Children's Hospital.

## Informed Consent

The authors declare that written informed consent was obtained from the patient's parents for publication of this case report.

## Author Contributions

GP, FdB, AM, and FL designed the study. GP, FdB, GC, DV, AntC, and IA cured the collection of the data. FdB, GC, IA, AndC, AM, GSC, TC, and FD-C interpreted and analyzed the data. GP, DV, FdB, and GC drafted the manuscript. FD-C performed immunohistochemistry analysis. EA performed molecular analysis. AM and FL critically revised the manuscript for intellectual content.

### Conflict of Interest Statement

The authors declare that the research was conducted in the absence of any commercial or financial relationships that could be construed as a potential conflict of interest.

## References

[1] De Graaf G Buckley F Skotko BG. Live births, natural losses, and elective terminations with Down syndrome in Massachusetts. Gene Med. (2016) 18:459–66. 10.1038/gim.2016.15 27126496

[2] Asim A Kumar A Muthuswamy S Jain S Agarwal S. down syndrome: An insight of the disease. J Biomed Sci. (2015) 22:1–9. 10.1186/s12929-015-0138-y 26062604 PMC4464633

[3] Carsetti R Valentini D Marcellini V Scarsella M Marasco E Giustini F. Reduced numbers of switched memory B cells with high terminal differentiation potential in down syndrome. Eur J Immunol. (2015) 45:903–14. 10.1002/eji.201445049 25472482 PMC4674966

[4] Weijerman ME van Furth AM Vonk Noordegraaf A van Wouwe JP Broers CJM Gemke RJBJ. Prevalence, neonatal characteristics, and first-year mortality of down syndrome: a national study. J Pediatrics. (2008) 152:15–9. 10.1016/j.jpeds.2007.09.045 18154890

[5] Hefti E Blanco JG. Pharmacokinetics of chemotherapeutic drugs in pediatric patients with down dyndrome and leukemia. J Pediatr Hematol Oncol. (2016) 38:283–7. 10.1097/MPH.0000000000000540.Pharmacokinetics 26907658 PMC4842084

[6] Dias-Santagata D Lam Q Vernovsky K Vena N Lennerz JK Borger DR. Braf V600E mutations are common in pleomorphic xanthoastrocytoma: diagnostic and therapeutic implications. PLoS ONE. (2011) 6:e017948: 10.1371/journal.pone.0017948 21479234 PMC3066220

[7] Schindler G Capper D Meyer J Janzarik W Omran H Herold-Mende C. Analysis of BRAF V600E mutation in 1,320 nervous system tumors reveals high mutation frequencies in pleomorphic xanthoastrocytoma, ganglioglioma and extra-cerebellar pilocytic astrocytoma. Acta Neuropathologica. (2011) 121:397–405. 10.1007/s00401-011-0802-6 21274720

[8] Lee EQ Ruland S LeBouef NR Wen PY. Successful treatment of a progressive BRAF V600E-mutated anaplastic pleomorphic xanthoastrocytoma with vemurafenib monotherapy. J Clin Oncol. (2016) 34:87–9. 10.1200/JCO.2012.48.0442 25092772

[9] Bruwier A Chantrain CF. Hematological disorders and leukemia in children with down syndrome. Eur J Pediatrics. (2012) 171:1301–7. 10.1007/s00431-011-1624-1 22113227

[10] Hasle H Haunstrup Clemmensen I Mikkelsen M. Risks of leukaemia and solid tumours in individuals with Down's syndrome. Lancet. (2000) 355:165–9. 10.1016/S0140-6736(99)05264-2 10675114

[11] Wechsler J Greene M McDevitt MA Anastasi J Karp JE Le Beau MM. Acquired mutations in GATA1 in the megakaryoblastic leukemia of down syndrome. Nature Gene. (2002) 32:148–52. 10.1038/ng955 12172547

[12] Ehara H Ohno K Ito H. Benign and malignant tumors in Down syndrome: Analysis of the 1514 autopsied cases in Japan. Pediatrics Int. (2011) 53:72–7. 10.1111/j.1442-200X.2010.03189.x 20573041

[13] Zhang AS Ostrom QT Kruchko C Rogers L Peereboom DM Barnholtz-Sloan JS. Complete prevalence of malignant primary brain tumors registry data in the United States compared with other common cancers, 2010. Neuro-Oncology. (2017) 19:726–35. 10.1093/neuonc/now252 28039365 PMC5464453

[14] Cairney CJ Sanguinetti G Ranghini E Chantry AD Nostro MC Bhattacharyya A. A systems biology approach to down syndrome: identification of notch/Wnt dysregulation in a model of stem cells aging. Biochim Biophys Acta. (2009) 1792:353–. 10.1016/j.bbadis.2009.01.015 19419698

[15] Gardiner K Costa ACS. The proteins of human chromosome 21. Am J Med Genet Part C Semi Med Gene. (2006) 142C:196–205. 10.1002/ajmg.c.30098 17048356 PMC3299406

[16] Gardiner K Davisson M. The sequence of human chromosome 21 and implications for research into down syndrome. Genome Biol. (2000) 1:0002. 10.1186/gb-2000-1-2-reviews0002 11178230 PMC138845

[17] Somasundar P McFadden DW Hileman SM Vona-Davis L. Leptin is a growth factor in cancer. J Surg Res. (2004) 116:337–. 10.1016/j.jss.2003.09.004 15013374

[18] Han T-J Wang X. Leptin and its receptor in hematologic malignancies. Int J Clin Exp Med. (2015) 8:19840–9. 26884894 PMC4723739

[19] Housa D Housov J Vernerov Z Haluzk M. Adipocytokines and cancer. Physiol Res. (2006) 55:233–244. 16238454 10.33549/physiolres.930848

[20] Kelesidis I Kelesidis T Mantzoros CS. Adiponectin and cancer: a systematic review. Br J Cancer. (2006) 94:1221–5. 10.1038/sj.bjc.6603051 16570048 PMC2361397

[21] Izadi V Farabad E Azadbakht L. Serum adiponectin level and different kinds of cancer: a review of recent evidence. ISRN Oncol. (2012) 2012:1–9. 10.5402/2012/982769 23213569 PMC3505647

[22] Boker LK Blumstein T Sadetzki S Luxenburg O Litvak I Akstein E. Incidence of leukemia and other cancers in Down syndrome subjects in Israel. Int J Cancer. (2001) 93:741–4. 10.1002/ijc.1383 11477589

[23] Da H Mortality and cancer incidence among individuals with Down syndrome. Arch Intern Med. (2003) 163:705–11. 10.1001/archinte.163.6.705 12639204

[24] Goldacre MJ. Cancers and immune related diseases associated with Down's syndrome: a record linkage study. Arch Dis Childhood. (2004) 89:1014–7. 10.1136/adc.2003.046219 15499053 PMC1719725

[25] Pátja K Pukkala E Sund R Iivanainen M Kaski M. Cancer incidence of persons with Down syndrome in Finland: a population-based study. Int J Cancer. (2006) 118:1769–72. 10.1002/ijc.21518 16231334

[26] Sullivan SG Hussain R Glasson EJ Bittles AH. The profile and incidence of cancer in Down syndrome. J Intellectual Disabi Res. (2007) 51:228–31. 10.1111/j.1365-2788.2006.00862.x 17300418

[27] Mercer ES Broecker B Smith EA Kirsch AJ Scherz HC Massad CA. Urological manifestations of Down syndrome. J Urol. (2004) 171:1250–3. 10.1097/01.ju.0000112915.69436.91 14767322

[28] Vachon L Fareau GE Wilson MG Chan LS. Testicular microlithiasis in patients with Down syndrome. J Pediatrics. (2006) 149:233–6. 10.1016/j.jpeds.2006.03.051 16887441

[29] Miller RW Relation between cancer and congenital defects in man. N Engl J Med. (1976) 295:87–93. 5327812 10.1056/NEJM196607142750208

[30] Porter KR McCarthy BJ Davis FG Kupelian V Freels S McCarthy B Prevalence estimates for primary brain tumors in the United States by behaviour and major histology groups. Neuro-Oncology. (2001) 3:152–8. 10.1215/S1522851700000569 11465395 PMC1920611

[31] Satgé D Stiller CA Rutkowski S Von Bueren AO Lacour B Sommelet D. A very rare cancer in Down syndrome: medulloblastoma. epidemiological data from 13 countries. J Neuro-Oncol. (2013) 112:107–14. 10.1007/s11060-012-1041-y 23307327

[32] Mangum R Varga E Boué DR Capper D Benesch M Leonard J. SHH desmoplastic/nodular medulloblastoma and Gorlin syndrome in the setting of Down syndrome: case report, molecular profiling, and review of the literature. Child's Nervous Syst. (2016) 32:2439–46. 10.1007/s00381-016-3185-0 27444290

[33] Yamamoto T Shinojima N Todaka T Nishikawa S Yano S Kuratsu JI. Meningioma in down syndrome. World Neurosurg. (2015) 84:866.E1–E6. 10.1016/j.wneu.2015.03.065 25862935

[34] Moore W Mathis D Gargan L Bowers DC Klesse LJ Margraf L. Pleomorphic xanthoastrocytoma of childhood: MR imaging and diffusion MR imaging features. AJNR Am J Neuroradiol. (2014) 35:2192–6. 10.3174/ajnr.A4011 24994821 PMC7965178

[35] Ida CM Rodriguez FJ Burger PC Caron AA Jenkins SM Spears GM. Pleomorphic xanthoastrocytoma: natural history and long-term follow-up. Brain Pathol. (2015) 25:575–86. 10.1111/bpa.12217 25318587 PMC4400218

[36] Pfister S Janzarik WG Remke M Ernst A Werft W Becker N. BRAF gene duplication constitutes a mechanism of MAPK pathway activation in low-grade astrocytomas. J Clini Invest. (2008) 118:1739–49. 10.1172/JCI33656DS1 18398503 PMC2289793

[37] Antonelli M Badiali M Moi L Buttarelli FR Baldi C Massimino M. *KIAA1549:BRAF* fusion gene in pediatric brain tumors of various histogenesis. Pediatr Blood Cancer. (2015) 62:724–7. 10.1002/pbc.25272 25382612

[38] Hsiao SJ Karajannis MA Diolaiti D Mansukhani MM Bender JG Kung AL. A novel, potentially targetable TMEM106B-BRAF fusion in pleomorphic xanthoastrocytoma. Cold Spring Harb Mol Case Stud. (2017) 3:a001396 10.1101/mcs.a001396 28299358 PMC5334470

[39] Nicolaides TP Li H Solomon DA Hariono S Hashizume R Barkovich K. Targeted Therapy for BRAFV600E malignant astrocytoma. Clin Cancer Res. (2011) 17:7595–604. 10.1158/1078-0432.CCR-11-1456 22038996 PMC3638050

[40] Chessells JM Harrison G Richards SM Bailey CC Hill FG Gibson BE. Down's syndrome and acute lymphoblastic leukaemia: clinical features and response to treatment. Arch Dis Child. (2001) 85:321–5. 10.1136/adc.85.4.321 11567943 PMC1718934

